# Including household effects in Big Data research: the experience of building a longitudinal residence algorithm using linked administrative data in Wales

**DOI:** 10.23889/ijpds.v3i1.452

**Published:** 2018-11-20

**Authors:** Karen Susan Tingay, Matthew Roberts, Charles BA Musselwhite

**Affiliations:** 1 Swansea University, Singleton Park, Swansea SA2 8PP

## Abstract

The effect of the wider social-environment on physical and emotional health has long been an area of study. Extrapolating the impact of the individual's immediate environment, such as living with a smoker or caring for a chronically-ill child, would potentially reduce confounding effects in health-related research. Surveys, including the UK Census, are beginning to collect data on household composition. However, these surveys are expensive, time consuming, and, as such, are only completed by a subsection of the population. Large-scale, linked databanks, such as the SAIL Databank at Swansea University, which hold routinely collected secondary use clinical and administrative datasets, are broader in scope, both in terms of the nature of the data held, and the population. The SAIL databank includes demographic data and a geographic indicator that makes it possible to identify groups of people that share accommodation, and in some cases the familial relationships among them. This paper describes a method for creating households, including considerations for how that information can be securely shared for research purposes. This approach has broad implications in Wales and beyond, opening up possibilities for more detailed population-level research that includes consideration of residential social interactions.

## Background

Our immediate physical, social and emotional environment impacts on our health and wellbeing. For example passive smoking has been linked to cancer (e.g. [1]); carrying the burden of responsibility for ill or disabled family members increased the risk of depression and anxiety in Greek caregivers [2]; frequent house moves or changes to the household composition have been connected to increased depression, emotional distress, and marijuana use in adolescents [3, 4], and in lower educational attainment in younger children [5]. Fowler, Henry & Marcal [6], found that unstable household composition can have long-standing impacts on mental health, lifestyle and antisocial behaviour in adolescence and early adulthood. Moreover, adverse life events experienced by other members of the family, such as racist abuse, illness or financial deprivation, have also been shown to increase an individual’s socio-emotional difficulties [7, 8].

In other respects, though, close social contact can be beneficial. Many studies have not only found an increase in survival time following diagnosis of cancer in married people compared with non-married patients (see, for example, [9, 10]), but also that married people tend to be diagnosed at an earlier stage than non-married patients [11]. Moreover, cohabitation with a significant other was a positive mitigating factor in survival of diseases, such as ovarian cancer [12].

It is clear from the above research that the immediate household environment can be seen as an important determinant of health and wellbeing. Surveys such as the UK Millennium Cohort Study [13], Add Health [14], and Understanding Society [15], as well as government-led surveys and censuses such as the Scottish [16] and Welsh [17] Health Surveys, have included questions about household composition and stability, but the ability to model this information using routinely collected health and administrative data sets is some way behind. Administrative datasets may be incomplete, particularly in transient populations, such as asylum seekers [18], or where data is context-specific [19]. Population-wide censuses, such as that conducted by the Office of National Statistics (ONS) in the United Kingdom (UK), are sufficiently financially costly and resource-intensive to only be carried out every ten years.

The ONS has done considerable work to construct households [20] using data from the dienally-collected census. Considerable interest in using administrative instead of census data resulted in a methodology for measuring household size and composition [21]. However, these estimates are currently based on 1% of addresses from existing 2011 Census data (the Population Coverage Survey (PCS)) [45] and only relate to snapshots in time [21]. For research purposes, a longitudinal view of household changes is desired. The ONS method also omits what are known as “complex addresses”, such as blocks of flats and other communal residence types. Again, these are households of interest for at least some research purposes, especially for research involving issues such as socioeconomic factors, or spread of infection.

There are plans announced to use linked data to construct households, especially in relation to validating the PCS estimates, but these are for England first before being tested in other UK nations. Given existing Welsh linked data, there is scope for Welsh household modelling to be conducted alongside ONS’s work, and to provide a dataset with a focus on research instead of population estimates.

While standardised terminologies such as Read and the Systematized Nomenclature of Medicine (SNOMED-CT) are widely used, many Electronic Health Records (EHRs) also use local codes that need to be mapped to a standardised terminology system in order to be linked with other EHRs of the same type [23]. Individuals must be anonymised in order to securely store their information, but this method must be robust enough to allow for datasets to link information on the same individual with few errors that might otherwise lead to spurious research results [24].

The ability to link records from different datasets, each containing a variety of different information, opens up huge possibilities for researchers, as well as challenges to keep data secure and non-identifiable. While there is increasing support for the benefits of data linkage for research purposes [25], aggregating individuals into groups risks increasing their identifiability. Given the well-known cost, relatively small sample size, and methodological issues of surveys [26], using routinely collected health and administrative datasets to produce household-level data appears to be an as yet untapped resource [27]. This article outlines the protocol for research modelling households using anonymised routine data. 

## Methods

### Design and conceptual framework

The Secure Anonymous Information Linkage (SAIL) databank, developed by the Swansea University Medical School, has collated routine clinical and administrative datasets from around Wales since 2006 [28]. At present, SAIL holds data on inpatient hospital admissions, outpatient hospital visits, birth and immunisation records, cancer screening, emergency department attendances, mortality records, congenital anomalies, and data from over 70% of GP practices in Wales, among other, more specific, datasets [29]. Although SAIL does not hold data from all GP practices in Wales, all Welsh residents and associated socio-demographic data are available through the Wales Demographic Service (WDS), which is the population spine for data linkage [30]. Individual-level data are anonymised using a split-file process, where identifiable information such as name, address, and date of birth, is separated from study data, such as GP events, and sent to a Trusted Third Party for anonymisation. An Anonymised Linkage Field (ALF) is sent to SAIL for probabilistic linkage with health datasets. This ALF is unique to each individual, allowing for information to be securely linked across different datasets.

**Figure 1: Data variables which can be used to identify people living at the same address:**
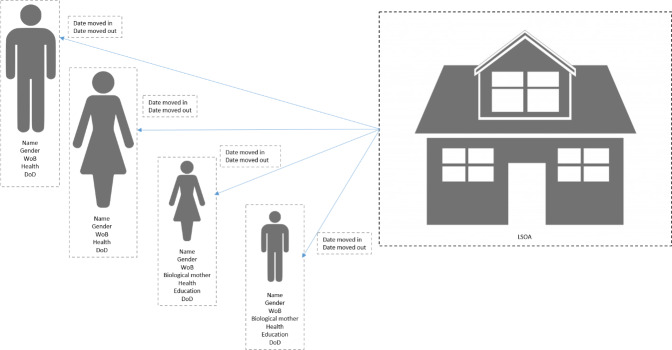


Each ALF is associated with a Residential Anonymised Record Linkage field (RALF, a de-identified address code based on the Unique Property Reference Number (UPRN) provided by the UK’s Ordnance Survey (OS). Figure 1 shows the types of data held in SAIL and how these could be used to link individuals to a household, as well as to answer health and wellbeing related research questions.

#### UK geographical coding

The OS is the UK’s national mapping agency, focusing on geographical surveying. The UPRN is a unique, linkable identifier for every British spatial address, which remains consistent across the life cycle of that address [31]. UPRNs are assigned by the governmental local authority responsible for that area, and form part of the National Address Gazetteer infrastructure, the UK address database of over 40 million addresses. UPRNs are allocated to new properties upon planning application approval, to sub-divided properties using a parent/child relationship of the subdivisions to the original (“parent”) UPRN, and to merged properties (i.e. where two properties are knocked together to make a single property). Upon demolition or merging, a UPRN is considered “historic” [32].

These UPRNs are de-identified as RALF codes, which are placed in relation to a Lower Super Output Area (LSOA) code, a computer-generated geographic area created by the ONS. LSOA’s are a sub-layer of the ONS population estimate hierarchical areas, known as Super Output Areas (SOAs), which have been in use UK-wide since the 2001 Census [32]. Each SOA level contains similar geographical and social populations and fit within existing government administrative boundaries.

The smallest SOA, Output Areas (OAs), consist of a minimum of 100 residents in 40 households, although up to 125 households was recommended where possible. In rural areas, such as parts of Wales, this minimum size is particularly important given the sparse population densities, although this does lead to some geographically large OAs compared to those in more urban areas.

LSOAs consist of between 4 and 6 OAs and cover an average of 1,500 residents in 650 households [33], typically with a minimum of 1,000 and a maximum of 3,000 residents. Approximately 4 to 5 LSOAs make up a Middle Super Output Areas (MSOAs), which hold an average of 7,200 residents (minimum of 5,000) in 2,000 households [34]. The minimum sizes for the ONS area hierarchies aim to ensure that confidentiality is maintained while allowing for population estimates to be calculated [32].

In the 2016 ONS outputs, Wales contains 1,909 LSOAs in 22 areas, consisting of an average of 1,631 people (minimum 900, maximum 4,512) [34]. 

#### RALF linkage and maintenance

RALFs are linked to an individual’s ALF based on the person’s place of residence as recorded on their GP registration [35]. When an individual registers with a GP, their address is recorded for contact and identification purposes. While the address may be confirmed when the individual receives a prescription, or is referred for specialist care, the onus is on the patient to notify any changes in address while registered at that practice. In the UK, GP registration is on a location basis, with GPs only accepting patients who live within their practice boundary. If a patient moves out of the practice boundary, and notifies their GP of that fact, they risk being deregistered with that practice.

Despite the risk of inaccurate address data, this linkage between the RALFs and LSOAs allows geographical outputs to be mapped, while retaining individual-level anonymity. To date, RALFs have been used to analyse a variety of environmental factors, such as access to alcohol outlets [36], vaccination uptake [37], fuel poverty [38], and housing regeneration [39].

### Development method

The ONS Census (2011) defines a household as "one person living alone or in a group of people (not necessarily related) living at the same address with common housekeeping - that is, sharing either a living room or sitting room or at least one meal a day" (pg. 2) [40]. Purely using routine administrative data, it is not possible to use the 2011 Census definition of a household, as existing routine datasets do not record whether household members share common housekeeping. Instead, a modified definition is proposed: "one person living alone or a group of people (not necessarily related) living at the same address as defined by the same UPRN". As the RALF for each resident contains the individual’s moving in and out date, the possibility exists to group people living at a RALF at the same time. Using privacy-preserving methods, this project will use RALFs and moving dates as a base to create Household Anonymised Linkage Fields (HALFs) of individuals living at the same residence during the same time period. A set of rules will be created which define the creation and completion of households.

These HALFs will be validated using existing survey data that contain questions on household composition. As the time periods in which individuals living at a residence are likely to overlap, it is likely that further HALF iterations will be required, e.g. HALF 1.1, 1.2... 1.n. These iterations can be used as a measure of the stability of the household. Such versioning allows for both longitudinal modelling, in which changes can be measured as they occur, and cross-sectional modelling, where the state of the household is measured at a specified point in time, either as a date or as a life stage.

Households may further be divided into biological (or partly biological) and non-biological. For children born in Wales, the ALF of the biological mother is recorded. These Maternal ALFs make it possible to infer mothers living with their biological children. While it is not possible, presently, to identify a biological father, an adult male living in the same HALF as children from or prior to the birth of the child/ren could be used to infer, if not a biological relationship, then a significant relationship to both adult male and child/ren. Stable households which move to different RALFs, either as full households or, for example, two adults moving repeatedly together, can be implied as a "family". Part of this project will address the amount of time a household unit remains stable in order for members to impact on each other.

By keeping the ALF, RALF, Maternal ALF, and HALF, individual effects within a household can be measured, through a “target” individual or person type that is the focus of study, e.g. children, or individuals with particular diagnoses. At the same time, different households can be studied, such as single-parent families, non-familial households, students living in university halls accommodation, or residents of a care facility. The Maternal ALF field allows extended families to be studied, and using the RALF and associated data allows for the geographical distance between families to be included, which could be an indicator of family support. Work has already been completed on a family identifier that allows both resident and non-resident family members to be identified via the Maternal ALF [41].

### Challenges in identifying families and households

One of the challenges in identifying family groups from routine clinical data, whether biological or sociological, is that electronic record systems are geared at individuals, rather than at the wider familial context. While there is growing recognition of the importance of collecting family history (see, for example, [42]), it is also recognised that current EHR designs put the onus for collecting this information on the patient [43].

Data quality is a further challenge. The dates recorded for an individual's residence in a RALF are based on their registration at a GP practice. However, not all of the GP practice registrations are up to date. Welsh Government figures report that 3,197,633 individuals were registered with a Welsh GP in 2016 [44]. Mid-year estimates for that same year give a population total of only 3,113,150 individuals [34], suggesting that some individuals may not have de-registered from their old practice, or even that some are registered with multiple practices. GP registration relies on the patient to provide accurate information. Pharmacies and specialist health services require a current address for the purposes of posting appointment or test letters, or to confirm identity. Furthermore, parents may register their children before themselves. As a result, a child may be seen to be living at a RALF before any adults [45]. These data quality challenges will be addressed further in a separate article.

### Validation

In this project, validation takes the algorithmic definition of “..the degree to which a model is an accurate representation of the real world from the perspective of its intended uses” [46, pg 6562]. In this instance, the best “real world” representation is the 2011 UK Census. The Census data are currently not available at the individual level in a way that would make it linkable to the routine data in SAIL, but aggregates of household sizes by LSOA are freely available from the ONS.

The HALF identifier will be measured against linked survey responses for different population groups, including children, and older adults. These surveys have been selected for their target populations, inclusion of household environment questions, and longitudinal nature. The latter will allow for the comparison of the identifier at different life stages. Where possible, survey respondents will be linked to their RALFs and HALFs at the time of the survey. This point prevalence will allow for direct comparisons between the survey and routine data. To allow for variations in population sizes as a whole, and any data issues beyond our control, the model will be considered valid if the results are within the 95% confidence level [47].

Variations in household composition between the data sources may show strengths and limitations of both methods of data collection. Where direct linkage is not possible, for example for aggregated results such as in the ONS Census, aggregated point prevalence comparisons will be drawn using the date of the data collection. As with the direct comparisons, there is likely to be some degree of variability between the observed and predicted data, requiring the use of 95% confidence levels.

Further detail on validation methods will be published separately.

### Ethical approval and considerations

The more information collected on an individual, the greater the chance of inadvertent re-identification, even by trained researchers. Grouping individuals for whom so much is known only increases this risk. While SAIL currently employs policies to minimise accidental disclosure [48], these have not yet been tested on aggregated groups in this way. In order to apply privacy-preserving research controls, one must first understand the nature of the problem. Analyses will be performed to identify epidemiological areas of small numbers that can then be used to create a set of rules by which household-level data can be released. It may be, for example, that Lower Super Output Area (LSOA) level information would prove too disclosive for some research questions, and that MSOA or Health Board geographic divisions would be more appropriate. Equally, individual responses to living with a household member with a rare condition may lead to too-small numbers for analysis, and aggregating to household-level responses may allow for more rigorous methodology. This is likely to need to be addressed on a project-by-project basis.

## Discussion

The option to include household-level details would add a level of complexity currently missing from Big Data studies. This would allow for a better understanding of deprivation and other factors, which could potentially impact on health and wellbeing. Studying the genome is an established research field, and advances in GIS methods and data capture is allowing the study of green and blue spaces on health and wellbeing (see, for example, [49, 50, 51]). By comparison, household effects, while known to be influential on population health, are lacking in Big Data research. That this is probably due to the current lack of available methodologies outside cohort studies, making this project, and others like it, all the more relevant to health and wellbeing research.

As well as for research, the model is also relevant to policy makers, who could make decisions based upon a more sophisticated portfolio of evidence than is available with existing methods. While there are known methodological issues around the existing data, not to mention unknown unknowns which will become apparent as the work progresses, the creation of such a tool promises to open up a range of research possibilities to researchers. While the identifier is being developed in Wales, the RALF and HALF methodologies could be applied throughout the UK and indeed to other nations with similar census type measures.

It is important to note the distinction between a research dataset and population statistics. While the UK’s ONS work on household estimates will be of tremendous value to policy makers, there are questions about its utility as a research tool. The lack of longitudinal reporting, inability to link to other data sources, such as health and education, and omission of communal residences limit its usefulness in health and social science research. In contrast, the model we are building includes household history, including following household members across different addresses, and changes to existing households over time. We have already conducted work into communal residences and are able to classify RALFs which are student halls of residence, residential care homes, and blocks of flats [52]. More work is required to refine the algorithms, however, particularly in relation to residential care homes and identifying households within blocks of flats.

The rules established by this project are likely to be relevant to any models developed by the ONS and other government household projects. Issues such as how households are defined using routine data, the strength of any mother-child linkages, multi-generational households, longitudinal and/or transient households, non-familial households, and communal households are common across residency datasets. These methods, while using a country-specific dataset, are likely to be of interest to international researchers and population data scientists interested in replicating building households from administrative data in their local jurisdiction. Such research opens up the possibility for more detailed population-level research through the analysis of residential social interactions.

## Declarations

This study was approved by SAIL’s independent Information Governance Review Panel (IGRP, project 0495).

### Authors’ contributions

KT conceptualized the study and its design and drafted the manuscript. MSR and CBAM contributed to the conception and design of the study. All authors reviewed the manuscript and approved it for publication.

### Availability of data and materials

The data supporting this study are available to access within the SAIL Databank and ADRC-W.
